# EST-SSR Markers’ Development Based on RNA-Sequencing and Their Application in Population Genetic Structure and Diversity Analysis of *Eleusine indica* in China

**DOI:** 10.3390/cimb45010011

**Published:** 2022-12-26

**Authors:** Jingchao Chen, Hailan Cui, Hongjuan Huang, Shouhui Wei, Yan Liu, Haiyan Yu, Yan Ma, Xiangju Li, Xiaoyan Ma

**Affiliations:** 1 State Key Laboratory for Biology of Plant Diseases and Insect Pests, Institute of Plant Protection, Chinese Academy of Agricultural Sciences, Beijing 100193, China; 2 Environment and Plant Protection Institute, Chinese Academy of Tropical Agricultural Sciences, Haikou 571101, China; 3 Institute of Cotton Research, Chinese Academy of Agricultural Sciences, Anyang 455000, China

**Keywords:** *Eleusine indica*, microsatellite markers, population structure, RNA-Seq

## Abstract

Goosegrass (*Eleusine indica*) is one of the worst agricultural weeds in China. Molecular markers were developed for genetic diversity and population structure analyses. In this study, we identified 8391 expressed sequence tag-simple sequence repeat (EST-SSR) markers from the de novo assembled unigenes of *E. indica.* Mononucleotides were the most abundant type of repeats (3591, 42.79%), followed by trinucleotides (3162, 37.68%). The most dominant mononucleotide and trinucleotide repeat motifs were A/T (3406, 40.59%) and AAT/ATT (103, 1.5%), respectively. Fourteen pairs of EST-SSR primers were verified and used to analyze the genetic diversity and population structure of 59 goosegrass populations. A total of 49 alleles were amplified, with the number of alleles (*N_a_*) ranging from two to eleven per locus, and the effective number of alleles (*N_e_*) ranged from 1.07 to 4.53. The average polymorphic information content (*PIC*) was 0.36. Genetic structure analysis (K = 2) and principal coordinate analysis divided 59 *E. indica* populations into two groups in a manner similar to the unweighted pair-group method (Dice genetic similarity coefficient = 0.700). This study developed a set of EST-SSR markers in *E. indica* and successfully analyzed the diversity and population genetic structures of 59 *E. indica* populations in China.

## 1. Introduction

Goosegrass (*Eleusine indica*), an annual weedy grass, is widely distributed in tropical and subtropical regions and has been reported to affect 46 crops which compete for nutrients, water, and light in 60 countries. This troublesome C4, self-fertilization weed has a 5–20 cm tall tufted stem, 2–3 columns of compound spike, and an ability to produce up to 140,000 seeds per plant [1]. Seedling emergence of goosegrass can reach up to 95% and germination was tolerant of abiotic stress such as salinity (up to 50 mM NaCl), temperature (up to 100 °C), and pH (5 to 10) [2]. These traits make it a strong competitor in infested areas which can lead to yield reductions of 20–50% for crops [3]. Moreover, this weed has recently been documented to occur in dry-seeded rice fields in 20 countries and in wet-seeded rice fields in another two countries [4]. Considering the ability of goosegrass to grow in such varied environments, there is a need for a better understanding of the genetic diversity of the various ecotypes to design effective practical tools and methods for goosegrass management.

Genetic diversity and population structure analyses have been used to assess potential evolutionary adaptations to changing environmental conditions in weeds [5]. They are important methods used to design long-term management strategies for weed infestations. Simple sequence repeats (SSRs) are widely used molecular markers for genetic diversity studies because they are co-dominant, highly polymorphic, reproducible, and highly accessible [6]. SSR markers are also employed to assess cultivar identification, DNA fingerprints, quantitative trait locus (QTL) mapping, and molecular-assisted selection (MAS), as they are cost-effective [7]. Transcriptome sequencing is an economical method and provides good resources for research related to gene expression, single nucleotide polymorphisms (SNPs), and SSRs [8].

Twenty-three SSR markers and twenty-four morphological traits were selected, and the phylogenetic relationships between 77 *Echinochloa* populations were successfully classified [9]. Likewise, 12 SSR markers were used to assess the genetic variation in 46 *Commelina communis* populations [10]. The development of de novo transcriptome sequencing technology was followed by the development of expressed sequence tag (EST)-SSR, which originates from the coding segment of DNA and provides new methods for analyzing population genetic structure and diversity [11]. Through RNA-seq technology, EST-SSRs have been utilized and developed in a variety of plant species, such as *Pinus koraiensis*, *Curcuma alismatifolia*, and *Crataegus pinnatifida* [12,13,14].

In China, goosegrass is widely distributed in orchards and vegetable gardens and among some general field crops such as corn (*Zea mays* L.), soybeans (*Glycine max* (L.) Merr.), and cotton (*Gossypium hirsutum* L.) [15,16]. The chemical control was the main strategy for goosegrass management, such as ACCase inhibitors, EPSPS inhibitors, and GS inhibitors [17]. However, the intense use of herbicide to control goosegrass results in the evolved resistance of the population [18]. The excessive use of synthetic herbicide for goosegrass control also leads to the problem of damaging soil microecology and environmental pollution [19]. The flexible growing potential of goosegrass makes it essential for us to gain a better understanding of the genetic diversity of various ecotypes. This study is part of an effort to (1) explore the EST-SSR markers for goosegrass based on published data obtained from RNA-seq in our previous study, (2) screen SSR markers with high polymorphism for goosegrass, and (3) analyze the genetic diversity of 59 goosegrass populations collected from 10 provinces in China using EST-SSRs. Moreover, our results indicate that the genetic diversity of goosegrass populations in these 10 provinces is relatively lower, and that next-generation sequencing techniques are powerful tools for SSR development and genetic analysis.

## 2. Materials and Methods

### 2.1. Transcriptome Data of E. indica

The transcriptome data on *E. indica* used in this study were obtained from our previous study, in which they were used to analyze differentially expressed genes [20]. Clean transcriptome data for *E. indica* have been uploaded to the Sequence Read Archive (SRA) public database (No. PRJNA323986). 

### 2.2. Plant Culture and DNA Extraction 

**Figure 1:**
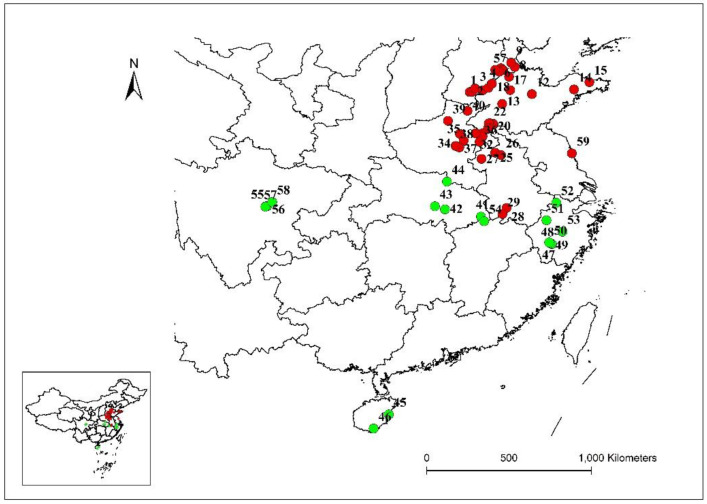
Distribution of the *E. indica* populations which collected seeds from 10 provinces in China. Each population was numbered and the color of the circle according to the results of different analysis in this research.

Seeds of 59 different goosegrass populations were collected from 10 provinces in China, and at least 50 individuals were selected and mixed thoroughly for each population (Figure 1, Table S1). To break dormancy, the seeds of different populations were soaked in a gibberellin solution (1000 mg·L^−1^) for 24 h and then sown in plastic pots (7.0 cm in diameter) that were filled with sand and soil (3:1 [*v*/*v*]), with no other seeds present. The plants were cultured in greenhouses under (20 ± 3) °C/(15 ± 3) °C day/night conditions until they reached the three-leaf stage. The seedlings were then collected, snap-frozen in liquid nitrogen, and stored at −80 °C [21]. Genomic DNA was extracted from ten individuals for each population using the Plant Genomic DNA Kit (TIANGEN Biotech (Beijing) Co., Ltd., Beijing, China), following the manufacturer instructions. The integrity and concentration of the DNA were determined using 1% agarose 1× Tris-Acetate-EDTA (TAE) gels stained with ethidium bromide [22]. The qualified total DNA obtained from each sample was diluted to the desired working concentration (25 ng μL^−1^) and stored at −20 °C until use.

### 2.3. EST-SSR Locus Screening and Primer Design

The Perl script MIcroSAtellite identification tool (MISA, http://pgrc.ipk-gatersleben.de/misa/misa.html (accessed on 10 February 2022)) was utilized to detect potential SSRs in assembled unigenes from *E. indica* [23]. Similar to other studies, the identification standards for the SSR motif contained minimum repeats of ten mononucleotides, six dinucleotides, and five tri-, tetra-, penta-, and hexanucleotide motif repeats [12]. Different primer pairs for each SSR locus were designed using Primer3 software (http://primer3.sourceforge.net/releases.php (accessed on 10 February 2022)) [24]. At least three primer pairs were selected for each SSR locus using the following criteria: primer length ranging from 18 bp to 22 bp (20 bp was the optimum length), melting temperature (Tm) between 55 °C and 65 °C (60 °C was the optimum annealing temperature), and PCR product size between 100 and 500 bp.

### 2.4. Primer Selection and Validation

To select high-quality primer pairs, they were designed from sequences containing tri-, tetra-, and penta-motif repeats. The sizes of the primers ranged from 19 to 21 bp, with similar guanine–cytosine contents (40–60%) and identical annealing temperatures (60 °C). PCR was performed using the designed primer pairs in a total volume of 20 μL, which included 1 μL of template DNA, 10 μL 2 × Taq PCR MasterMix (with green dye) (TIANGEN Biotech (Beijing) Co., Ltd., Beijing, China), 1 μL of primer (10 μM per type), and 7 μL double-distilled water [12]. The PCR amplification conditions included an initialization step at 94 °C that lasted for 3 min, followed by 30 cycles of heating to 94 °C for 30 s. Then, the reaction was heated to 60 °C for 30 s and then to 72 °C for 20 s. A final extension occurred over the course of 10 min at 72 °C. The products for all the primers were examined on a 2% agarose gel, and primers that displayed obvious bands of expected sizes were selected for subsequent analysis.

### 2.5. PCR Amplification and Capillary Electrophoresis

After all the selected primers were designed, an M13 tail was added to the 5′ end of forward primers. The M13 universal primer randomly labeled with different fluorescent dyes (TAMRA [yellow], HEX [green], ROX [red], and FAM [blue]) was added for multiplexed PCR, which was conducted in a 20 μL reaction [14]. This included 10 μL 2 × Taq PCR MasterMix (TIANGEN Biotech (Beijing) Co., Ltd., Beijing, China), 2 μL template DNA, 0.2 μL M13-tailed forward primer, 0.2 μL of reverse primer, 0.4 μL of fluorescently labeled M13 primer, and 7.2 μL double-distilled water. The PCR amplification conditions were as follows: heating to 94 °C for 3 min, 15 heating cycles at 94 °C for 40 s, then at 60 °C for 30 s, and, finally, at 72 °C for 1 min. This was followed by 25 cycles of heating at 94 °C for 40 s, then at 53 °C for 30 s, and, finally, at 72 °C for 1 min. The last extension occurred at 72 °C over the course of 10 min [25]. Amplified PCR products were detected using an ABI PRISM 3730XL DNA Analyzer (Applied Biosystems, Foster, CA, USA). Allelic sizes were recorded automatically using individual GeneScan files. Sizes and peaks were calibrated automatically against the ROX-500 size standards.

### 2.6. Statistical Analyses

The degree of polymorphism for each primer pair was determined by their polymorphism information content (*PIC*) values, which were calculated using MicroSatellite tools (MS tools). The POPGENE32 (version 1.31) was selected to calculate the parameters of population genetics for each primer pair, including the number of alleles per locus (*Na*), observed heterozygosity (*Ho*), expected heterozygosity (*He*), and the effective number of alleles (*Ne*) [10,14]. NTsys-pc software (version 2.10s) was used to calculate the Dice genetic similarity coefficient values via the unweighted pair-group method, with arithmetic averaging (UPGMA) cluster analysis [10]. GeneAlEx (version 6.5) was applied to estimate the molecular variance (AMOVA) in populations, in populations within groups, and among groups. Additionally, principal component analysis (PCA) was performed using GeneALEX (version 6.5). The population genetic structures of 59 *E. indica* populations were analyzed using STRUCTURE software (version 2.3.4), and 20 independent runs were performed for each K value. There were 100,000 burn-in period iterations and 500,000 Markov chain Monte Carlo repetitions per run (K ranged from 1 to 10) [14].

## 3. Results

### 3.1. Frequency and Distribution of EST-SSRs

In total, 8391 EST-SSR loci were detected among the 14,364 examined unigenes (Table 1). Among the detected EST-SSRs, the mononucleotide was the most abundant type of repeat (3591, 42.79%).

This was followed by the trinucleotide (3162, 37.68%), the dinucleotide (1520, 18.11%), the tetranucleotide (84, 1.00%), the pentanucleotide (17, 0.20%), and, finally, the hexanucleotide (7, 0.08%). The frequencies of EST-SSRs for different tandem repeats were detected, and results showed that the largest number of tandem repeats was 5 (2188, 26.08%), followed by 10 (2085, 24.85%), 6 (1268, 15.11%), and 11 (779, 9.28%) (Table 2). 

Additionally, A/T was the most abundant motif (3406, 40.59%), followed by CCG/CGG (1241, 14.79%) and AG/CT (1053, 12.55%), which accounted for 67.93% of the total number of SSRs (Table 2). 

### 3.2. Polymorphism Analysis of Selected SSR Markers

The 14 EST-SSR markers selected by PCR were successfully amplified across all 59 samples and showed high polymorphism. In total, 35 alleles were detected using these markers. The number of alleles per sample (*N_a_*) ranged from two to eleven, with an average of 3.50, and the effective number of alleles per sample (*N_e_*) varied from 1.07 to 4.53, with a mean value of 1.94 (Table 3). Shannon’s information index (*I*) ranged from 0.17 to 1.78. The observed heterozygosity (*H_o_*) ranged from 0.33 to 0.98. The gene diversity (expected heterozygosity [*H_e_*]) ranged from 0.07 to 0.78, and the lowest and highest values of polymorphism information content (*PIC*) were 0.05 and 0.56, respectively, with an average value of 0.36 (Table 3).

### 3.3. Genetic Structure and Principal Component Analysis

**Figure 2:**
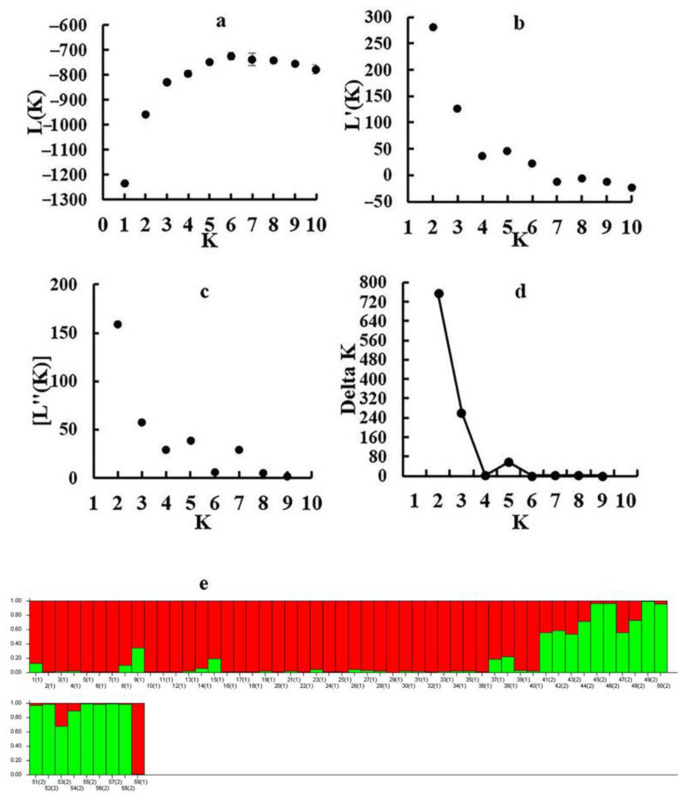
Results of STRUCTURE analysis for 59 populations using 14 EST-SSR markers: (**a**) Plot mean likelihood L(K) and variance per K value. (**b**) Plot of mean rate of change of the likelihood distribution L’(K) per K value. (**c**) Plot of absolute value of the second order rate of the change of the likelihood distribution |L’’(K)| per K value. (**d**) Estimation of population using Delta K value with cluster K ranging from 1 to 10. (**e**) Estimation of population structure based on STRUCTURE analysis.

**Figure 3:**
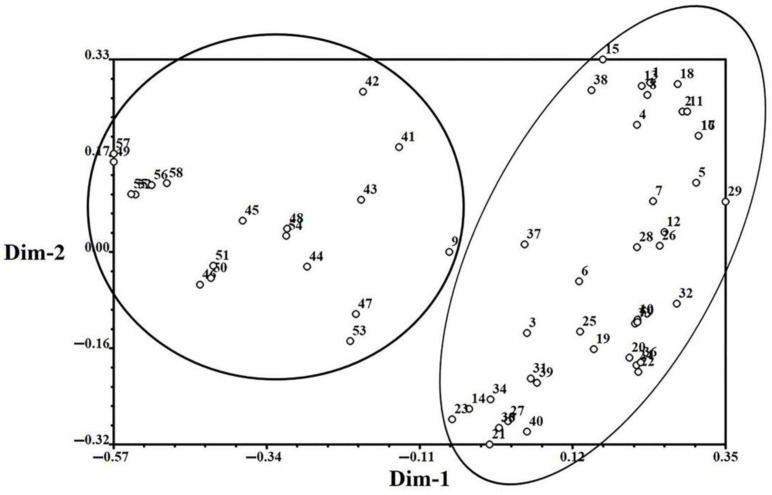
Principal component analysis based on 14 EST-SSR markers in *E. indica*. Coordinate 1 explained 33.80% of the total variation and Coordinate 2 explained 15.53% of the total variation.

The population genetic structures of detected individuals from 59 populations were analyzed using STRUCTURE 2.3.4. In this study, the number of clusters was set from 1 to 10, with 20 repetitions per run. The results showed that the optimal K value was observed at K = 2, using the maximum Delta k value. All collected individuals were then further divided into two groups. Group I contained 41 sites from five provinces (Hebei, Shandong, Henan, Anhui, and Jiangsu), and Group II contained 18 sites from five provinces (Zhejiang, Sichuan, Hainan, Hubei, and Jiangxi) (Figure 2). Population genetic structure analysis was conducted using PCA, and the individuals were grouped into two groups based on the first two principal coordinates. Coordinate 1 explained 33.80% of the total variation and Coordinate 2 explained 15.53% of the total variation (Figure 3).

### 3.4. Cluster Analysis

**Figure 4:**
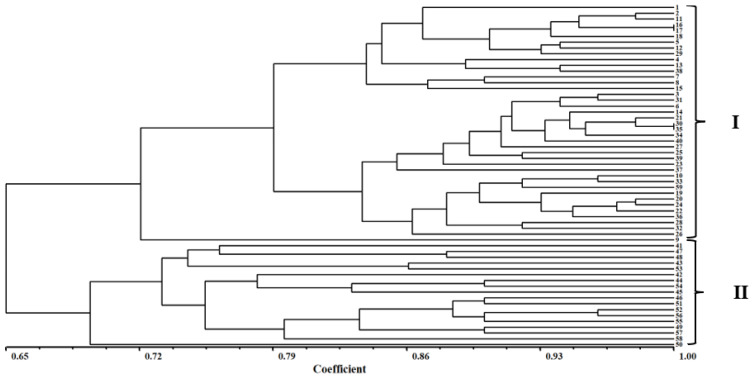
UPGMA analysis of 59 populations of *E. indica* by the Sequential Agglomerative Hierarchical and Nested Clustering (SAHN) module of the NTSYS-pc. The FIND module was used to identify all trees that could result from different choices of tied similarity or dissimilarity values.

The genetic similarity coefficient ranged from 0.70 to 1.00 and was based on the Dice similarity coefficient (which was analyzed by NTsys-pc software). Genetic relationships were elucidated using a dendrogram based on SSR data. Based on the cluster analysis, the 59 *E. indica* populations could be divided into two major groups (I–II) when the Dice genetic similarity coefficient was 0.70. Group I clustered 41 populations from five provinces (Hebei, Shandong, Henan, Anhui, and Jiangsu), and Group II clustered 18 populations from five provinces (Zhejiang, Sichuan, Hainan, Hubei, and Jiangxi) (Figure 4). 

AMOVA values showed that a large portion of the variation is attributed to “among populations”. It was shown that 40% of the total variation came from groups clustered using the UPGMA method. A total of 42% of the total variation was due to differences between populations; nevertheless, 18% was attributed to differences between the individuals within the populations (Table 4).

## 4. Discussion

A limited number of EST sequences are available for use with goosegrass, although screening genic SSR markers utilized in genetic diversity studies via RNA-seq technology has become one of the most efficient methods [26,27]. In this study, 6849 out of 48,852 unigenes contained SSRs, representing approximately 14.02% of the transcriptomic sequences for goosegrass. This frequency was significantly higher than that of Torreya grandis (2.7%) [28] but lower than that of *Posidonia oceanica* (17.5%) [29]. The SSR frequency varies widely between different species and may be affected by SSR search criteria, unique species properties, and the size of the unigene assembly dataset [30]. In our study, six different repeat motifs were identified, and mononucleotides repeats were the most frequent repeats (42.79%), followed by trinucleotides (37.68%). In contrast, tetr- (1.00%), penta- (0.20%), and hexanucleotide repeats (0.08%) were shown to be less frequent. Most studies have found that mono-, di-, and trinucleotide repeats occur at relatively high frequencies, while other types of nucleotide repeats are rare [10,31,32]. As shown in Table 2, similar to most plant species, A/T repeats in goosegrass were significantly more abundant than G/C repeats [33,34]. The two most abundant trinucleotide repeat motifs in this study were CCG/CGG (14.79%) and AGG/CCT (6.79%). Consistent with our results, many studies have revealed that the CCG/CGG type is the most abundant trinucleotide repeat in monocots but is rarely found in dicotyledonous plants [30,31].

In this study, the degrees of the polymorphism of different loci for 59 goosegrass populations were measured based on PIC values, which can be used to assess our ability to detect molecular marker polymorphisms. The results showed that locus SSR216 had the highest genetic diversity, with *He* and *PIC* values of 0.78 and 0.48, respectively. The average *PIC* value for all loci was 0.36, and ten had values greater than 0.35. The high repeat number in the dinucleotide locus of SSR216, which was higher than that for the trinucleotide repeats, may be one of the reasons for the greatest diversity, according to a previous assumption [35]. These results indicated the presence of a moderate level of genetic diversity of most loci within their populations, with the SSR224 locus being known to have high polymorphism when its *PIC* value became greater than 0.56. Yang et al. found that the *PIC* values for developed markers in *C. communis* did not exceed 0.50, which is similar to our findings. The *PIC* value was often higher than 0.50 in some industrial crops, such as *Pinus koraiensis*, *Crataegus pinnatifida*, and *Curcuma alismatifolia* [12,13,14]. Goosegrass is a self-pollinated weed species, a trait that affects the level of genetic diversity within the species as a whole, and when the self-crossing rate increases, population genetic diversity tends to decrease [12].

The population genetic structure was analyzed according to the structure that was divided by the 59 populations into two groups. The PCA results were consistent with the results of the genetic structural analysis performed using the same EST-SSR markers and individuals. Gene flow is an important factor affecting the genetic structure of plant population structure. Based on the structure result, most of the populations in group I were from north China to the Yangtze River, while most populations in group II were from south China to the Yangtze River. A similar result was also found in *C. communis*, from which 46 populations were divided into three major groups using 12 SSR markers [10]. Our data further suggested that geographical location might play a more important role than other factors in these populations.

## 5. Conclusions

In this study, we developed and analyzed a set of EST-SSR markers derived from the *E. indica* transcriptome. A total of 14 EST-SSR primer markers were verified, and the population genetic structure and diversity of 59 *E. indica* populations, which were divided into two groups, were successfully analyzed. These results indicate that next-generation sequencing techniques are powerful tools for SSR development and genetic analysis. 

## Figures and Tables

**Table 1:** Summary of analyses of expressed sequence tag-simple repeat (EST-SSRs) in *E. indica*.

Item	Parameters	Number
EST-SSR	Total number of sequences examined	48852
	Total size of examined sequences (bp)	41400899
	Total number of identified SSRs	8391
	Number of SSR containing sequences	6849
	Number of sequences containing more than 1 SSR	1206
	Number of SSRs present in compound formation	451

**Table 2:** Frequencies of different repeat motifs in SSRs in *E. indica*.

Repeats	5	6	7	8	9	10	11	12	13	14	15	16+	Total	Percentage
A/T	-	-	-	-	-	1791	632	285	190	110	63	335	3406	40.59
C/G	-	-	-	-	-	43	38	26	9	14	14	41	185	2.20
AC/GT	-	93	36	39	23	22	24	3	-	-	-	1	241	2.87
AG/CT	-	283	174	149	147	214	80	5	-	-	-	1	1053	12.55
AT/AT	-	70	22	20	10	14	5	2	-	-	-	0	143	1.70
CG/CG	-	73	6	4	-	-	-	-	-	-	-	0	83	0.99
AAC/GTT	52	29	13	2	-	-	-	-	-	-	-	0	96	1.14
AAG/CTT	143	54	36	6	-	-	-	-	-	-	-	0	239	2.89
AAT/ATT	21	9	12	3	-	-	-	-	-	-	1	0	46	0.55
ACC/GGT	140	57	27	2	-	-	-	-	-	-	-	0	226	2.69
ACG/CGT	136	41	9	4	-	-	-	-	-	-	-	0	190	2.26
ACT/AGT	21	3	5		-	-	-	-	-	-	-	1	30	0.36
AGC/CTG	257	96	27	2	-	-	-	-	-	-	-	0	382	4.55
AGG/CCT	334	151	78	6	-	-	-	1	-	-	-	0	570	6.79
ATC/ATG	90	30	19	3	-	-	-	-	-	-	-	0	142	1.69
CCG/CGG	896	264	76	5	-	-	-	-	-	-	-	0	1241	14.79
Others	98	15	1	1	0	1	0	0	0	0	1	1	118	1.41
Total	2188	1268	541	246	180	2085	779	322	199	124	79	380	8391	100

**Table 3:** Characteristics of 14 selected EST-SSR markers in *E. indica*.

Primer Name	Repeat	Primer Sequence (5′-3′)	TM (°C)	*Na*	*Ne*	*Ho*	*He*	*I*	*PIC*
SSR10	(TTTG)5	AACCAGTTCTTCCTCTGCCG GCCAGCACACCACTCATTTG	60	2.00	1.68	0.95	0.40	0.59	0.44
SSR12	(ATCC)5	TCCTCCTCCTCTGCCCTTTT GCATCCCACCGAACACACTA	60	3.00	1.82	0.97	0.45	0.75	0.42
SSR27	(CGAT)5	GGCTGCTGATGCTTAAACGG TCTAGCTGAGGCAGGACAGT	60	2.00	1.83	0.95	0.45	0.65	0.40
SSR104	(GAG)5	GGGCTCTAGGGACTACACCA GGCTTTCAGAAGGGCTGCTA	60	3.00	1.07	0.98	0.07	0.17	0.07
SSR105	(CCG)5	CGACCACGAGTTCTGCTTCT CCCGCCCTCCAATTTCTCTT	60	3.00	1.23	0.86	0.19	0.40	0.05
SSR137	(ATGT)5	CTGTCTCTGCCCTCCAACAG GGTAGCGTCCAGGATCATGC	60	2.00	2.00	0.95	0.50	0.69	0.45
SSR138	(TATC)5	TAACAGCGACCGCATCTACC AACCTCGCCGTTGTTCAGAG	60	3.00	1.76	0.92	0.43	0.69	0.35
SSR142	(TTTC)5	ACACTCACTCCCTGATCCCT CGGAGGCCCACGTTTCTTAT	60	2.00	1.59	0.95	0.37	0.56	0.32
SSR154	(ATTT)5	CGCGCGCATTTTCATCAGAT CTTGGGATGCTCGTAGCCAT	60	2.00	1.96	0.95	0.49	0.68	0.44
SSR186	(ATGT)5	CTGTCTCTGCCCTCCAACAG GGTAGCGTCCAGGATCATGC	60	2.00	2.00	0.93	0.50	0.69	0.43
SSR202	(GAG)12	CTCCACCATCTCCTTCCTCG CACAAGAAGATCCCCGTGCT	60	4.00	1.38	0.92	0.28	0.51	0.19
SSR204	(TCTCTT)10	GCAGCAAGCCCATGATCTTG TCAGCAGCTGAGCTTACTCC	60	5.00	2.19	0.88	0.54	1.03	0.47
SSR216	(AT)12	CGGTGCGTGACAGTCAAAAG GCCTCCCTGATCCGTTCATC	60	11.00	4.53	0.33	0.78	1.78	0.48
SSR224	(TCAG)15	TGGCTTACCAACAGGCACAA AACAAACAACGCGTCTTGGC	60	5.00	2.20	0.97	0.55	1.09	0.56
Mean				3.50	1.94	0.89	0.43	0.74	0.36
St. Dev				2.41	0.82	0.17	0.17	0.38	0.15

Note: *N_a_*: Observed number of alleles; *N_e_*: effective number of alleles; *H_o_*: observed heterozygosity; *H_e_*: expected heterozygosity; *I*: Shannon’s information index; *PIC*: polymorphism information content.

**Table 4:** Analysis of molecular variance (AMOVA) among *E. indica* populations and clustered groups.

Source of Variation	*d.f.*	MS	SS	%Total	Est. Var.
Among groups	1	82.427	82.427	40	1.568
Among populations	57	3.987	227.276	42	1.638
Total	58		351.703	82	3.205

*d.f*., degree of freedom; MS, mean squared deviations; SS, sum of squares; Est. Var., estimate variance; Prob, the significance of the variance components after 999 random permutations. Values among groups, among populations and within populations are considered highly significant (*p* < 0.001).

## Data Availability

Clean transcriptome data for *E. indica* can be uploaded to the Sequence Read Archive (SRA) public database (No. PRJNA323986) in NCBI.

## Supplementary Materials

The following supporting information can be downloaded at: https://www.mdpi.com/article/10.3390/cimb45010011/s1, Table S1: The information for the populations of *E. indica*.
