# Supplementary material for: EST-SSR Markers’ Development Based on RNA-Sequencing and Their Application in Population Genetic Structure and Diversity Analysis of Eleusine indica in China
Source: Curr Issues Mol Biol. 2022 Dec 26;45(1):141–50. doi: 10.3390/cimb45010011 (PMC9856800; doi:10.3390/cimb45010011)
Supplement: Supplementary file 1 [file cimb-45-00011-s001.zip › Table S1.pdf]

Tables S1. The information for the populations of *E. indica*			
**Province**	**Site number**	**Northern latitude**	**East longitude**
Hebei	1	36.78405556	115.00175
Hebei	2	36.801	115.1422778
Hebei	3	36.98322222	115.2938056
Hebei	4	38.08841667	116.7086111
Hebei	5	37.98141667	116.8261389
Hebei	6	37.90191944	116.6194389
Hebei	7	38	116.3997556
Hebei	8	38.15712222	117.4713333
Hebei	9	38.38793611	117.2704417
Shandong	10	36.88770278	117.2161472
Shandong	11	36.88558056	115.7137333
Shandong	12	36.67508333	118.4045556
Shandong	13	36.14025	116.7806944
Shandong	14	36.93072222	120.7046111
Shandong	15	37.32858333	121.5429167
Shandong	16	37.012	116.0156389
Shandong	17	37.62188889	117.15375
Shandong	18	37.24497778	116.220675
Shandong	19	35.02186111	116.30625
Shandong	20	35.05272222	116.2796667
Shandong	21	35.07325	116.1304722
Shandong	22	35.07255556	116.0433889
Shandong	23	34.83277778	116.0388611
Shandong	24	34.79963889	115.9993333
Anhui	25	33.46947222	116.3944722
Anhui	26	33.3415	116.7208611
Anhui	27	33.13416667	115.6463889
Anhui	28	30.45158333	117.01975
Anhui	29	30.11947222	116.7890833
Henan	30	34.53191667	115.7074444
Henan	31	34.35894444	115.7074444
Henan	32	34.53327778	115.3244444
Henan	33	34.50213889	114.4623056
Henan	34	33.84252778	114.2363611
Henan	35	34.13105556	114.6699444
Henan	36	34.06183333	115.54475
Henan	37	33.76391667	114.4357778
Henan	38	33.84180556	114.2365
Henan	39	35.20866667	113.8156944
Henan	40	35.76098333	114.8826028
Hubei	41	29.97686111	115.6213611
Hubei	42	30.378	113.6515833
Hubei	43	30.54163889	113.1007778
Hubei	44	31.89019444	113.7574722
Hainan	45	19.16725278	110.5785417
Hainan	46	18.39470524	109.7518992
Zhejiang	47	28.56856667	119.3468
Zhejiang	48	28.48243333	119.5013

**Zhejiang**	**49**	**28.51694083**	**119.40935**
**Zhejiang**	**50**	**28.56856667**	**119.3468**
**Zhejiang**	**51**	**29.77651667**	**119.19985**
**Zhejiang**	**52**	**30.73668333**	**119.7327**
**Zhejiang**	**53**	**29.15608333**	**120.0785278**
**Jiangxi**	**54**	**29.72711111**	**115.7918611**
**Sichuan**	**55**	**30.53882**	**103.849541**
**Sichuan**	**56**	**30.54916667**	**103.9320556**
**Sichuan**	**57**	**30.52025**	**103.8403611**
**Sichuan**	**58**	**30.77870882**	**104.2098985**
**Jiangsu**	**59**	**33.43541667**	**120.5851667**
